# Oxidative Stress and DNA Damage Markers in Colorectal Cancer

**DOI:** 10.3390/ijms231911664

**Published:** 2022-10-01

**Authors:** Delia Acevedo-León, Lidia Monzó-Beltrán, Laura Pérez-Sánchez, Eva Naranjo-Morillo, Segundo Ángel Gómez-Abril, Nuria Estañ-Capell, Celia Bañuls, Guillermo Sáez

**Affiliations:** 1 Servicio de Análisis Clínicos, Hospital Universitario Dr. Peset-FISABIO, 46017 Valencia, Spain; 2 Departamento de Bioquímica y Biología Molecular, Facultad de Medicina y Odontotología-INCLIVA, Universidad de Valencia, 46010 Valencia, Spain; 3 Servicio de Cirugía General y Aparato Digestivo, Hospital Universitario Dr. Peset-FISABIO, 46017 Valencia, Spain; 4 Servicio de Endocrinología y Nutrición, Hospital Universitario Dr. Peset-FISABIO, 46017 Valencia, Spain

**Keywords:** colorectal cancer, oxidative stress, catalase, glutathione, 8-oxodG, F2-Isoprotanes, tumor markers, inflammatory profile

## Abstract

Oxidative stress (OS) and inflammation are known to play an important role in chronic diseases, including cancer, and specifically colorectal cancer (CRC). The main objective of this study was to explore the diagnostic potential of OS markers in patients with CRC, which may translate into an early diagnosis of the disease. To do this, we compared results with those in a group of healthy controls and assessed whether there were significant differences. In addition, we explored possible correlations with the presence of tumors and tumor stage, with anemia and with inflammatory markers used in clinical practice. The study included 80 patients with CRC and 60 healthy controls. The following OS markers were analyzed: catalase (CAT), reduced glutathione (GSH) and oxidized glutathione (GSSG) in serum; and 8-oxo-7,8-dihydro-2′-deoxyguanosine (8-oxodG) and F2-isoprotanes in urine (F2-IsoPs). Tumor markers (CEA and CA 19.9), anemia markers (hemoglobin, hematocrit and medium corpuscular volume) and inflammatory markers (leukocytes, neutrophils, N/L index, platelets, fibrinogen, C-reactive protein, CRP and IL-6) were also determined. Comparison of means between patients and controls revealed highly significant differences for all OS markers, with an increase in the prooxidant markers GSSG, GSSG/GSH ratio, 8-oxodG and F2-IsoPs, and a decrease in the antioxidant markers CAT and GSH. Tumor and inflammatory markers (except CRP) correlated positively with GSSG, GSSG/GSH ratio, 8-oxodG and F2-IsoPs, and negatively with CAT and GSH. In view of the results obtained, OS markers may constitute a useful tool for the early diagnosis of CRC patients.

## 1. Introduction

Colorectal cancer (CRC) is a very prevalent tumor and one of the main causes of cancer-related death in industrialized countries, according to data from Globocan 2020 [1]. It is now the most commonly diagnosed cancer in both men and women in the world, with an estimated 1.9 million cases per year (10% of the total number of cancer cases), third in incidence after breast and lung cancer. In 2020, CRC produced 0.9 million deaths worldwide (9.4% of deaths from cancer). By 2040, these numbers are projected to rise to 1.92 million new cases of colon cancer and 0.7 million new cases of rectal cancers [2]. 

The five-year survival rate for people with colorectal cancer is 65%, though it can vary depending on several factors, particularly tumor stage. The degree of invasion of a patient’s tumor is the most important factor in determining the stage of CRC (TNM classification), and varies from carcinoma in situ (stage 0) to distant extension of the tumor to different parts of the body (stage IV). The five-year survival rate of people with localized stage colorectal cancer is 91%, but only approximately 37% of patients are diagnosed at this early stage. If the cancer has spread to distant parts of the body, the five-year survival rate is 15% [3].

Reactive oxygen species (ROS) are generated by the biochemical redox reactions that occur as a part of normal cell metabolism, but high doses and/or inadequate elimination of ROS result in oxidative stress (OS), which can cause severe metabolic malfunctions and cell damage [4]. High levels of ROS react with biomolecules, such as lipids, nucleic acids and proteins, and undermine their function [5]. 

OS and inflammation are known to play an important role in chronic diseases, including cancer [6,7,8], and specifically CRC [9,10]. On the other hand, high cellular proliferation in a microenvironment rich in inflammatory cytokines fuels the development of cancer. This process is ROS-dependent and has been implicated in the initiation and progression of CRC [11]. 

In the genome, guanine (G) is highly susceptible to OS due to its lower redox potential. A typical lesion formed under oxidative conditions is 8-oxo-7,8-dihydro-2′-deoxyguanosine (8-oxodG), a mutagenic base. If not repaired adequately, 8-oxodG can pair with adenine (A) to cause GC > TA transversion mutations [12]. In this way, 8-oxodG is one of the primary forms of free radical-induced oxidative DNA damage and is, thus, a pivotal marker of the initiation and promotion of carcinogenesis in humans [13].

F2-IsoPs, cyclic peroxides of the eicosanoid family, are a product of the oxidation of essential fatty acids composed by 20 carbon atoms, and are isomers of prostaglandins. However, unlike prostaglandins, which are produced as a direct action of the enzyme cyclooxygenase, F2-IsoPs are a final product of the peroxidation of essential fatty acids (mainly arachidonic acid) induced by ROS. These molecules are both biomarkers and mediators of OS in numerous disease settings [14,15].

In physiological conditions, antioxidant systems exist in equilibrium with pro-oxidant systems. Protective antioxidant mechanisms include catalase (CAT) and the glutathione system. Physiologically, CAT plays a fundamental role in the detoxification of hydrogen peroxide produced by some enzymes located in peroxisomes, such as amino-oxidase. In cells, glutathione is generally found in a reduced state (GSH) and, to a lesser extent, in an oxidized state (GSSG). This is a result of the glutathione reductase enzyme reducing GSSG back to GSH. The enzyme is constitutively active and inducible in OS situations. Some studies of CRC have reported a decrease of GSH [16,17] and a decrease of CAT [18].

Several reports have documented the importance of OS as a major etiological factor in colorectal carcinogenesis and the role of antioxidants in countering OS and preventing the occurrence of CRC. However, few studies to date have linked OS levels to common clinical markers of tumor progression. We hypothesized that pro-oxidant markers are increased and antioxidant markers are decreased in CRC patients with respect to healthy controls. Therefore, we set out to assess OS markers in patients with CRC and to compare them with those in a group of healthy controls. In addition, we explored possible correlations of OS markers with the presence of tumors, with anemia and with inflammatory markers currently used in clinical practice. We also analyzed OS marker values according to tumor stage with the purpose of obtaining information that could aid early diagnosis of the disease.

## 2. Results

### 2.1. Clinical and Anthropometric Characteristics

This study included a total of 80 patients with CRC and 60 controls. Significant differences were observed between the two groups in terms of weight and height, and, consequently, in body mass index (BMI) and age. Thus, a univariate analysis of variance was performed with BMI and age as covariates, in order to eliminate potentially confounding effects (Table 1). 

CRC patients displayed significantly higher levels of glucose, albumin, transferrin and IL-6, and lower total cholesterol, HDL-cholesterol, LDL-cholesterol, ferritin, iron and transferrin saturation index than controls. However, no differences were detected regarding creatinine, urea, glomerular filtration, triglycerides or proteins. Although significant differences in all the parameters of iron metabolism and hemoglobin were observed between CRC patients and controls, values of hemoglobin, hematocrit and MCV did not indicate the presence of anemia. In addition, iron metabolism was altered among our patients, with a lower iron, ferritin and transferrin saturation index in 52.5% of patients versus controls. In terms of inflammatory profile, significant differences were observed between the two groups in all markers, except for lymphocytes and C-reactive protein (CRP). In addition, levels of CEA and CA 19.9 tumor markers were significantly higher in CRC patients vs. controls. A total of 24 patients (30%) presented elevated levels of CEA and 14 patients (17.5%) presented elevated levels of CA 19.9.

### 2.2. Oxidative Stress Markers

**Figure 1:**
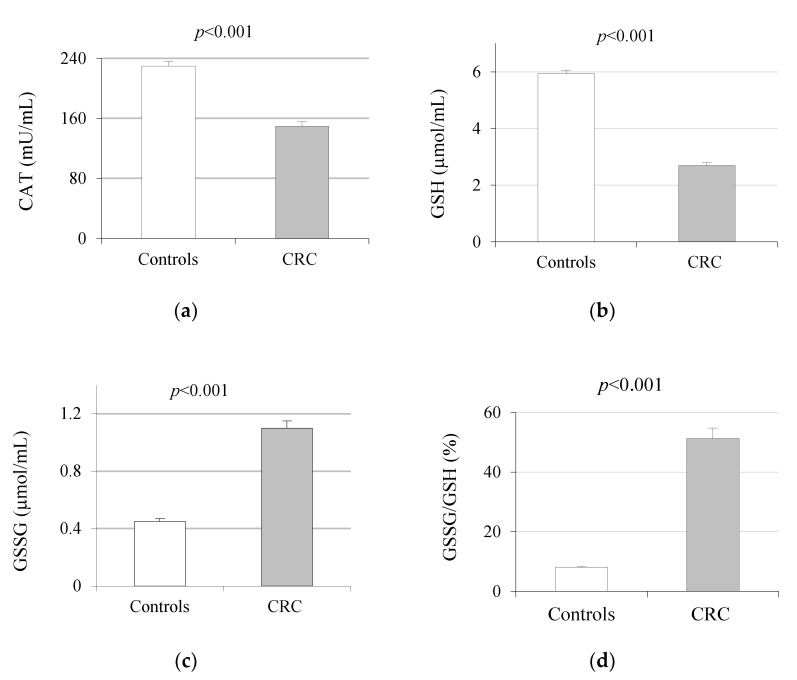

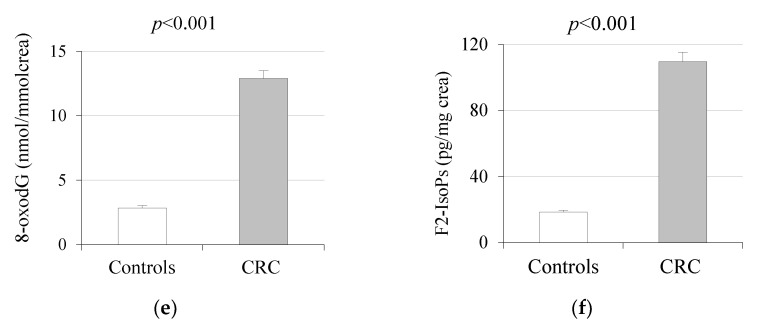
Levels of serum catalase (**a**); reduced glutathione (**b**); oxidized glutathione (**c**); GSSG/GSH ratio (**d**); and urine 8-oxo-7,8-dihydro-2′-deoxyguanosine (**e**) and F2-IsoProstanes (**f**) in controls and CRC patients; *p*-value adjusted for age and body mass index. Data are expressed as mean ± standard error. CAT: catalase; GSH: reduced glutathione; GSSG: oxidized glutathione; 8-oxodG: 8-oxo-7,8-dihydro-2′-deoxyguanosine; F2-IsoPs: F2-IsoProstanes.

The means of the OS markers differed significantly between the control group and CRC patients, even after adjustment for age and BMI covariates. Serum CAT and GSH levels were significantly lower in CRC patients with respect to controls (Figure 1). Conversely, serum levels of GSSG and urine 8-oxodG and F2-IsoPs levels were higher in the CRC vs. control group.

### 2.3. Association with Tumor Stages

The degree of invasion of the patient’s tumor was classified according to the TNM system. The number of patients per TNM stage was as follows: Stage 0: 8 (10%); Stage I: 17 (21.3%); Stage II: 20 (25%); Stage III: 26 (32.5%) and Stage IV: 9 (11.3%). These stages were regrouped for the correlation study into qualitative variables 0, 1 and 2. Thus, the patient tumors were distributed as follows: localized (Stage 0) = 44 patients (55.7%); progressive (Stage 1) = 26 patients (32.9%); and invasive (Stage 2) = 9 patients (11.4%).

#### 2.3.1. Biochemical, Anemia, Tumor and Inflammation Markers

We explored possible associations of the biochemical, anemia and inflammation markers with tumor stage, but found none, with the exception of ferritin, which was considerably higher in stage 2 than in stages 0 and 1 (Supplementary Table S1).

Although CEA increased in stage 2 compared to stages 0 and 1, the difference was not significant. In contrast, the increase in CA 19.9 did show significant differences in stage 2 (Supplementary Table S1).

#### 2.3.2. Oxidative Stress Markers

When OS markers were analyzed according to tumor stages, significant differences were observed in all cases, particularly in stage 2 (advanced). CAT and GSH were significantly lower in stage 2 than in stages 0 and 1, while levels of GSSG, 8-oxodG and F2-Isoprostanes, and GSSG/GSH ratio, were far higher in stage 2 than in stages 0 and 1 (Table 2).

### 2.4. Correlation Analysis

The correlation analysis revealed that both TM CEA and CA 19.9 correlated negatively with CAT and GSH and positively with GSSG, GSSG/GSH ratio, 8-oxodG and F2-IsoPs. N/L ratio, platelets, fibrinogen and IL-6 correlated with all the OS markers, while CRP did not correlate with any marker. In contrast, the anemia markers did not correlate with OS markers (Table 3). 

## 3. Discussion

Our data indicate that the means of all the OS markers analyzed differed in a highly significant manner between CRC patients and controls. This difference remained significant after adjustment for age and BMI, which was surprising given that OS is reported to increase in obesity [19,20,21]. The means of CAT and GSH were lower in CRC patients than in controls, whereas those of the rest of the pro-oxidant and DNA damage markers (GSSG, 8-oxodG and F2-IsoPs) were higher.

Various enzymes are responsible for eliminating excess ROS formation in cells. Cecerska-Heryće et al. studied antioxidant enzymes in CRC patients and reported an increased expression of superoxide dismutase (SOD), while the expression of CAT, glutathione reductase and glutathione peroxidase was decreased [22].

In line with our results, decreased CAT levels have previously been reported in serum and tumor tissue in colon adenocarcinomas and other cancers [23,24,25]. In a study of 36 patients with CRC and 40 healthy controls aged between 38 and 82 years (similar to the age range of our patients), Chang et al. observed lower levels of serum antioxidant enzymes and GSH and increased levels of serum 8-oxodG in patients with respect to controls [17]. In contrast, other authors have reported an imbalance of antioxidant enzymes in patients with CRC, with decreased CAT levels and increased levels of some enzymes in the serum, such as SOD [26].

In general, any condition associated with excess ROS can result in a drop of serum GSH levels. In line with the present results, Baltruskeviciene et al. [27], in a study of 40 healthy controls and 58 CRC patients, found serum GSH to be significantly lower in the latter. Dusak et al. also reported decreased levels of GSH in the serum of 25 CRC patients with respect to healthy controls [16]. Serum levels of GSH and GSSG and the GSSG/GSH ratio have been pointed to as potentially effective TMs, not only in CRC, but in other types of cancer [28]. Rasool et al., in a study of 50 CRC patients and 20 controls, detected lower levels of CAT and GSH in the serum of patients with respect to controls, which, once again, is in accordance with our results [18]. Similarly, other studies have reported decreased levels of GSH and CAT in tumor tissue [27,29].

Excessive ROS production causes damage to ADN and can initiate the carcinogenesis process in several types of cancer [12,30]. Another OS marker whose levels in urine are elevated in different types of cancer is 8-oxodG [13,31]. Roszkowski et al. determined levels of 8-oxodG in plasma, serum and leukocytes of patients with CRC, and found them to be higher than in controls [32]. Measurement of markers in urine rather than tissue or lymphocytes offers advantages; it is a non-invasive method, and urine remains stable at −20 °C for a long period of time. In a recent review on the role of OS in cancer development, the authors focused on 8-oxodG, antioxidative enzymes and products of lipid peroxidation (malondialdehyde, MDA) as biomarkers of different types of cancer. In general, levels of antioxidative enzymes are lower in cancer, while 8-oxodG levels are higher [33]. Moreover, adherence to a Mediterranean diet has been associated with decreased levels of 8-oxodG in CRC [34], while synbiotic foods—which include prebiotics and probiotics, a combination of live bacteria and mostly carbohydrate-based substances, such as dietary fiber or starch—have been shown to promote the activity of beneficial bacteria in the gut. In this sense, perioperative administration of probiotics/synbiotics may help to decrease postoperative complications in CRC patients [35]. Indeed, several meta-analyses have pre-investigated the effects of probiotics/synbiotics on oxidative and antioxidant factors in serum, showing that supplementation can significantly increase total antioxidant capacity (TAC), and the GSH, MDA and nitric oxide (NO) levels in adults. Therefore, probiotic/synbiotic supplementation might be effective in reducing OS levels and, thus, in preventing cancer and other chronic diseases [36].

F2-IsoPs are considered by some authors to be the most reliable markers for monitoring OS in vivo, due to their high chemical stability and sensitivity to OS [37,38]. Indeed, Forman et al. argued that F2-IsoPs were the best currently identified marker of lipid peroxidation [39]. Other authors analyzed F2-IsoPs in serum and found significantly higher levels in patients with CRC than in controls [29]. Il’yasova et al. recommended 8-oxodG and F2-IsoPs as the best biomarkers with which to monitor oxidative status over time [40], while other authors have endorsed using a combination of both to study OS in different tumors [41,42].

Although elevated levels of both TM CEA and CA 19.9 are generally used in prognosis in clinical practice [43], a comparison with OS shows that their sensitivity is considerably low, especially in the case of CA 19.9 [28].

The tumor microenvironment, which is composed of macrophages, neutrophils and fibroblasts, plays a significant role in cancer progression [44]. In this context, several systemic inflammation-based prognostic parameters, such as neutrophil-lymphocyte ratio, modified Glasgow Prognostic Score and platelet-lymphocyte ratio, have been found to have prognostic value in a large number of studies and are now widely available in clinical laboratories worldwide [45]. The combination of preoperative serum CEA and CA 19.9 with peripheral blood routine indexes (NLR or neutrophil/lymphocyte ratio, MLR or monocyte/lymphocyte ratio, and PLR or platelet/lymphocyte ratio) can provide sound prognostic information in CRC patients [46]. 

In addition, the activation of inflammatory interleukins, such as IL-1 and IL-6, and the overexpression of membrane proteins, such as MAP17, play an important role in the development of chronic inflammation and its potential progression to cancer [47]. 

IL-6 was the best marker of inflammation in our patients, as it was higher in more than half of them (specifically, in 52.5%) and correlated with all the OS markers analyzed. When the mean baseline of our patients was compared with that of our healthy controls, the difference proved to be significant. This is consistent with the results of other researchers who have reported that IL-6 signaling contributes to CRC development and is a predictor of poor prognosis in CRC patients [48,49,50]. In our study, IL-6 was also associated with CEA.

We observed that inflammatory markers such as fibrinogen, platelets, neutrophils, leukocytes, N/L index and IL-6 correlated with all the OS markers analyzed. In contrast, CRP did not correlate with any OS marker. This is in line with several studies that have shown a lack of association of CRP levels with CRC risk or survival [50,51,52]. 

Anemia, defined as a decrease in hemoglobin in the blood, is a systemic inflammatory reaction and a hematological paraneoplastic syndrome due to tumor-generated substances that mimic or block normal endocrine signals for the development of the hematological lineage [53]. Mean hemoglobin values in our patients were lower than those in the control group, but did not reach the range of anemia. Moreover, iron metabolism was altered in patients, with a lower iron, ferritin and transferrin saturation index registered in 52.5% versus controls. In this context, in a study involving 429 CRC patients, Wilson et al. [54] detected iron deficiencies in 48% (similar to our study).

Several reports have related increased OS in CRC patients with a worse prognosis, especially in advanced stages [55,56,57]. A comprehensive study of 150 patients with CRC who underwent surgery analyzed OS parameters (MDA and 4-HNE) in peripheral and mesenteric blood according to four tumor stages (I-IV). The authors observed that levels of both OS markers increased with tumor stage [58], in line with previous results published by our group [28].

Other authors have studied the relationship of dietary, constitutional and genetic factors and different markers with tumor stage in CRC patients. Bomfim Gomes Campos et al. analyzed the association of inflammatory, anthropometric, functional and oxidative markers (MDA) with tumor stage in newly-diagnosed CRC patients and concluded that IL-6 and triceps skinfold were indicators of cancer stage. However, the study had several limitations; it was based on only 28 CRC patients, who were divided into two groups—I (Stages 0-III) and II (Stage IV)—and a healthy control group was not included [59].

In summary, the OS markers we have evaluated presented significant differences between CRC patients and controls, with lower levels of the antioxidant markers CAT and GSH, and higher levels of the pro-oxidant markers GSSG, 8-oxodG and F2-IsoPs, in the former when compared to the latter. Furthermore, in our CRC patients, the level of oxidation was higher in advanced stage 2 than in the more localized stages, which also suggests a strong relationship between OS and CRC. These results, together with the convenience of sample collection in the case of 8-oxodG and F2-IsoPs, endorse OS markers as a useful tool for the early diagnosis of CRC. Future studies measuring gene and protein expression in peripheral blood mononuclear cells or tumor tissue would undoubtedly complement our results.

## 4. Materials and Methods

### 4.1. Study Design

This was a longitudinal and prospective, observational study in patients diagnosed with colorectal tumor and who were candidates for tumor resection surgery and/or chemo-radiotherapy treatment at the General and Digestive Surgery Service of University Hospital Dr. Peset. A control group of age-matched healthy volunteers was included for comparison. A flow chart of the study protocol is provided as Supplementary Figure S1.

The study was designed in accordance with the principles of ethics of the Declaration of Helsinki (Finland, 1964), and was evaluated and approved by the Clinical Research Ethics Committee of University Hospital Dr. Peset. Informed consent was obtained from all the subjects involved in the study.

### 4.2. Study Population

A total of 80 patients with a diagnosis of colorectal tumor (both advanced adenomas and carcinomas) were enrolled in the study between March 2019 and January 2020. Advanced adenomatous neoplasia was confirmed when polyps reached 1 cm or more in diameter and had a villous component or high-grade dysplasia.

Patients of both genders, without comorbidities, and who had not received radiotherapy or chemotherapy, were eligible for inclusion in the study. CRC patients with systemic or autoimmune diseases (diabetes, insulin resistance, hypertension, coronary heart disease, rheumatoid arthritis and psoriasis), or lung, thyroid, liver, kidney, gastrointestinal or infectious diseases (chronic viral hepatitis and HIV infection), were excluded. Smokers and patients who had taken drugs (antibiotics, non-steroidal anti-inflammatory drugs, glucocorticoids, vitamins or dietary supplements) in the previous three months were also excluded from the study. In addition, patients who did not want to participate voluntarily in the study and those whose health could be compromised during the study due to advanced age or poor general health were excluded.

As a control population, 60 healthy subjects of both genders, with a BMI < 30 kg/m^2^, with clinical characteristics similar to those of the CRC patients, and no clinical pathologies (dyslipidemia, diabetes mellitus, arterial hypertension, chronic renal failure, ischemic heart disease or inflammatory bowel disease) were included in the study.

### 4.3. Analytical Assays

#### 4.3.1. Biochemical and Hematological Studies in Serum and Blood Samples

Serum samples were extracted after 12 h fasting and collected in dry 10 mL tubes with a silicone gel separator and coagulation accelerator. Following clot retraction (about 30 min, at room temperature) the samples were centrifuged at 3500× rpm for 5 min in a Rotina 380R Hettich centrifuge (Tuttlingen, Germany). Aliquots were separated from the serum for determination of OS markers, and a volume of 1 mL was retained for biochemical determinations, which were performed on the same day. The aliquots were frozen at −80 °C in the New Brunswick Scientific Premium U 410 freezer (Eppendorf, NJ, USA) until assays were performed.

A basic clinical analysis plus a study of ferric metabolism, inflammation markers and TM were requested for each serum sample. Metabolites were analyzed in an automated chain of Architect C16000 equipment from Abbott (Chicago, IL, USA) and in the Cobas 6000 from Roche Diagnostics (Manheim, Germany), in accordance with the spectrophotometric and immunochemical methodology of the manufacturers.

Analysis of biochemical parameters, including triglycerides, total cholesterol and HDL cholesterol, glucose, total proteins, albumin, urea, creatinine and electrolytes, was performed with automatic analyzers following standard procedures. Validation of appropriate internal controls was performed on every workday.

The glomerular filtration rate was estimated by means of the Chronic Kidney Disease Epidemiology Collaboration (CKD-EPI), a mathematical formula that includes serum creatinine, age, sex and race as variables.

For the hematimetric analysis, samples of whole blood were collected in EDTA-K3 tubes and assessed with a Beckman-Coulter LH 500 hematology analyzer (Brea, CA, USA). For fibrinogen analysis, samples of whole blood were collected in sodium citrate tubes and assessed with an ACL-TOP of Instrumentation Laboratory Company (Bedford, MA, USA).

#### 4.3.2. Oxidative Stress and Inflammatory Profile

For determination of OS markers, Cayman Chemical (Ann Arbor, MI, USA) spectrophotometric assays were employed, except for 8-oxodG, which was determined by High Performance Chromatography with Electrochemical Detection (HPLC-EC). A 515 HPLC Waters Pump (Milford, CT, USA) with ESA Coulochem II electrochemical HPLC detector (Hucoa-Erlöss, Madrid, Spain) was also employed.

CAT, GSH, GSSG and GSSG/GSH ratio were determined in serum, and 8-oxodG and F2-IsoPs in morning urine samples (the results were corrected with urinary creatinine to eliminate variability in the concentrations of the urine samples).

TM and IL-6 were determined by electrochemiluminescence using a Cobas 6000 from Roche Diagnostics (Mannheim, Germany). CRP was performed by spectrophotometry in the automated chain of Architect C16000 equipment from Abbott (Chicago, IL, USA). 

### 4.4. Statistical Analysis

Statistical analysis was performed with the Statistical Package for the Social Sciences (SPSS), version 17.0 for Windows (Chicago, IL, USA). The results of the continuous quantitative variables are expressed as means and standard deviation in the tables, and means and standard error of means in the figures. The Kolmogorov–Smirnov test was used to assess normality for continuous variables, and the data conformed to normal distribution patterns. The differences in means between the control group and CRC patients were compared using a Student’s *t* test for parametric samples or a Mann–Whitney U test for nonparametric samples. Spearman’s correlation coefficients were employed to measure the strength of the association between OS, tumor, anemia and inflammatory markers. 

To perform the statistical analysis of potential associations of the different variables with tumor stage, the AJCC TNM stages were redefined as qualitative variables 0, 1 and 2, as follows: tumors located in the colon/rectum, including TNM stages 0, I and II = Stage 0; progressive (regional) tumors with lymph node involvement, TNM stage III = Stage 1; and invasive (advanced) tumors, with distant metastasis or distant peritoneal sites, TNM stage IV = Stage 2. A one-way analysis of variance-ANOVA for independent samples was performed, followed by the Student–Newmann–Keuls (SNK) post hoc test to determine the mean difference of the studied markers between stages. In this case, we used Levene’s test to assess the equality of variances for parametric variables and the Kruskal–Wallis test for non-parametric variables. The level of statistical significance used in all cases was α, with significant differences confirmed when *p* < 0.05.

## Figures and Tables

**Table 1:** Demographic, anthropometric, biochemical, anemia, inflammatory and tumor parameters of controls and colorectal cancer (CRC) patients.

Variable	Control (*n* = 60)	CRC (*n* = 80)	*p*-Value	* Adjusted *p*-Value
Age (years)	64.0 ± 9.0	67.5 ± 11.8	0.052	-
Male/Female (n; %)	36/24; 60/40	52/28; 65/35	0.548	-
Weight (kg)	74.4 ± 16.3	77.3 ± 15	<0.001	-
Height (cm)	168 ± 11	165.5 ± 9.8	<0.001	-
BMI (kg/m^2^)	26.1 ± 3.0	28.1 ± 3.9	0.001	-
Glucose (mg/dL)	96.2 ± 14.4	116.6 ± 52.3	<0.001	0.001
Creatinine (mg/dL)	0.9 ± 0.2	2.0 ± 8.6	0.319	0.269
Urea (mg/dL)	40.9 ± 7.2	38.8 ± 15.9	0.878	0.296
EGF (mL/min)	81.1 ± 8.7	78.9 ± 20.9	0.399	0.720
Total cholesterol (mg/dL)	195.7 ± 34.3	180.4 ± 39.1	0.018	0.026
HDL cholesterol (mg/dL)	50.7 ± 12.8	43.2 ± 10.8	<0.001	<0.001
LDL cholesterol (mg/dL)	144.9 ± 30.0	114.5 ± 34.7	<0.001	<0.001
Triglycerides (mg/dL)	112.0 (98; 142.8)	108.5 (83.3; 141)	0.954	0.777
Uric acid (mg/dL)	4.5 ± 1.6	5.3 ± 1.7	0.003	0.058
Albumin (g/dL)	3.9 ± 0.4	4.2 ± 0.5	0.001	<0.001
Total proteins (g/dL)	7.0 ± 0.5	6.9 ± 0.4	0.472	0.577
Ferritin (µg/L)	133.5 ± 75.4	67.5 ± 144.9	0.002	0.008
Iron (µg/dL)	79.7 ± 19.1	57.6 ± 41.0	<0.001	<0.001
Transferrin (mg/dL)	269.3 ± 46.5	291.1 ± 51.4	0.011	0.016
TSI (%)	30.5 ± 8.9	16.2 ± 11.1	<0.001	<0.001
CRP (mg/L)	6.2 ± 1.3	11.0 ± 23.6	0.072	0.076
IL-6 (pg/mL)	2.7 ± 1.4	19.7 ± 26.5	<0.001	<0.001
Leukocytes (×10^3^/mm^3^)	7.0 ± 1.7	7.7 ± 1.8	0.016	0.018
Neutrophils (%)	56.0 ± 6.0	62.9 ± 8.4	<0.001	<0.001
Lymphocytes (%)	29.8 ± 10.2	27.7 ± 12.0	0.221	0.531
N/L (-)	2.1 ± 1.4	2.7 ± 1.6	0.005	0.013
Platelets (x10^5^/mm^3^)	206.0 ± 60.0	253.5 ± 74.0	<0.001	<0.001
Fibrinogen (mg/dL)	352.3 ± 70.8	483.8 ± 101.7	<0.001	<0.001
Hemoglobin (g/dL)	14.2 ± 1.5	12.9 ± 1.9	<0.001	<0.001
Hematocrit (%)	42.5 ± 4.6	37.7 ± 5.1	<0.001	<0.001
MCV (fL)	85.7 ± 4.8	83.4 ± 8.4	0.046	0.135
CEA (ng/mL)	2.35 ± 1.15	7.51 ± 11.0	<0.001	0.004
CA 19.9 (UI/mL)	9.03 ± 6.53	23.0 ± 30.4	0.002	0.011

* *p*-value adjusted for age and body mass index (BMI); n: number of cases; EGF: estimated glomerular filtration; TSI: transferrin saturation index; CRP: C-reactive protein; IL-6: interleukin 6; N/L: neutrophil/lymphocyte index; MCV: mean corpuscular volume; CEA: carcinoembryonic antigen; CA 19.9: carbohydrate antigen 19.9. Data are expressed as mean ± standard deviation. In the case of values that did not follow a normal distribution (triglycerides), the median (quartile 25/75) was used.

**Table 2:** Study of oxidative stress markers in CRC patients according to tumor stages.

Variable	Stage 0	Stage 1	Stage 2	*p*-Value
CAT (mU/mL)	160.1 ± 5.43 ^a^	140.0 ± 3.92 ^b^	88.9 ± 4.32 ^b^	0.002
GSH (µmol/mL)	2.52 ± 0.72 ^a^	2.32 ± 0.65 ^a,b^	1.86 ± 0.09 ^b^	0.045
GSSG (µmol/mL)	1.06 ± 0.44 ^a^	1.03 ± 0.39 ^a^	1.68 ± 1.68 ^b^	0.002
GSSG/GSH (%)	47.6 ± 31.3 ^a^	48.3 ± 25.7 ^a^	90.7 ± 33.9 ^b^	0.002
8-oxodG (nmol/mmol crea)	12.2 ± 4.35 ^a^	12.0 ± 3.97 ^a^	19.3 ± 6.36 ^b^	0.001
F2-IsoPs (pg/mg crea)	106.6 ± 3.8 ^a^	107.2 ± 3.5 ^a^	128.2 ± 6.3 ^b^	0.004

Results are expressed as mean ± standard error. Levels of catalase, reduced glutathione and oxidized glutathione are measured in serum, and 8-oxo-7,8-dihydro-2′-deoxyguanosine and F2-IsoProstanes in urine. Values with different superscript letters (a, b) were significantly different when the 3 groups were compared by one-way ANOVA followed by a Student–Newman–Keuls post hoc test. CAT: catalase; GSH: reduced glutathione; GSSG: oxidized Glutathione; 8-oxodG: 8-oxo-7,8-dihydro-2′-deoxyguanosine; F2-IsoPs: F2-Isoprostanes.

**Table 3:** Correlation between oxidative stress and tumor and inflammatory markers in controls and CRC patients.

	CAT (mU/mL)	GSH (μmol/mL)	GSSG (μmol/mL)	GSSG/GSH (%)	8-oxodG (nmol/mmol crea)	F2-IsoPs (pg/mg crea)
Tumor markers						
CEA (ng/mL)	−0.379 ***	−0.270 **	0.292 **	0.276 **	0.300 ***	0.183 **
CA 19.9 (IU/mL)	−0.412 ***	−0.292 **	0.345 ***	0.322 ***	0.257 **	0.343 ***
Anemia markers						
Hemoglobin (g/dL)	n.s.	n.s.	n.s.	n.s.	n.s.	n.s.
Hematocrit (%)	n.s.	n.s.	n.s.	n.s.	n.s.	n.s.
MCV (fL)	n.s.	n.s.	n.s.	n.s.	n.s.	n.s.
Inflammatory markers						
CRP (mg/L)	n.s.	n.s.	n.s.	n.s.	n.s.	n.s.
IL-6 (pg/mL)	−0.237 ***	−0.328 ***	0.419 ***	0.385 ***	0.366***	0.367 ***
Leukocytes (×10^3^/mm^3^)	−0.237 **	n.s.	0.186 *	0.173 *	0.193 *	0.260 **
Neutrophils (%)	−0.236 **	−0.318 ***	0.362 ***	0.330 ***	0.332 ***	0.356 ***
N/L (-)	−0.187 *	−0.175 *	0.232 **	0.181 *	0.246 **	0.268 ***
Platelets (×10^5^/mm^3^)	−0.256 **	−0.300 ***	0.240 **	0.239 **	0.302 ***	0.294 ***
Fibrinogen (mg/dL)	−0.471 ***	−0.462 ***	0.521 ***	0.521 ***	0.522 ***	0.535 ***

Data are expressed as Spearman’s correlation coefficient (r) with statistical significance (* *p* < 0.05; ** *p* < 0.01; *** *p* < 0.001) for each pair of variables. When the correlation is not significant, it is represented as n.s. CAT: catalase; GSH: reduced glutathione; GSSG: oxidized glutathione; 8-oxodG: 8-oxo-7,8-dihydro-2′-deoxyguanosine; F2-IsoPs: F2-IsoProstanes; CRP: C-reactive protein; IL-6: interleukin 6; N/L: neutrophil/lymphocyte ratio; CEA: carcinoembryonic antigen; CA 19.9: carbohydrate antigen 19.9.

## Data Availability

The data presented in this study are available on request from the corresponding author.

## Supplementary Materials

The following supporting information can be downloaded at: https://www.mdpi.com/article/10.3390/ijms231911664/s1.
