# Supplementary material for: Oxidative Stress and DNA Damage Markers in Colorectal Cancer
Source: Int J Mol Sci. 2022 Oct 1;23(19):11664. doi: 10.3390/ijms231911664 (PMC9569897; doi:10.3390/ijms231911664)

Supplementary Table S1. Study of biochemical, inflammation, anemia and tumor markers in CRC patients according to tumor stages.

Variable	Stage 0	Stage 1	Stage 2	*p*-value
Biochemical markers				
Glucose (mg/dL)	115.3±28.9	117.8±37.5	122.9±55.8	0.835
Total cholesterol (mg/dL)	181.3±37.1	179.9±45.5	183.5±26.1	0.972
HDL cholesterol (mg/dL)	44.0±11.1	43.1±10.7	40.6±10.4	0.709
LDL cholesterol (mg/dL)	114.7±33.4	115.0±38.1	115.3±34.7	0.999
Uric acid (mg/dL)	5.57±1.67	5.09±1.53	4.94±2.28	0.402
Albumin (g(dL)	4.23±0.57	4.14±0.33	4.16±0.32	0.725
Inflammation markers				
IL-6 (pg/mL)	14.0±20.3	28.5±34.1	20.7±20.4	0.079
CRP (mg/L)	6.61±3.76	16.6±38.6	15.6±12.7	0.189
Leukocytes (x10^3/mm^3)	7.37±1.76	7.98±1.56	8.55±2.05	0.123
Neutrophils (%)	62.9±7.64	62.4±9.33	64.5±8.45	0.835
N/L (-)	2.53±0.82	2.80±2.49	3.09±1.41	0.618
Platelets (x10^5/mm^3)	242.0±66.1	273.5±87.7	253.4±54.7	0.217
Fibrinogen (mg/dL)	465.3±90.8	504.8±107.2	514.0±133.7	0.195
Anemia markers				
Hemoglobin (g/dL)	12.4±1.96	12.1±1.80	11.7±1.40	0.647
Hematocrit (%)	38.0±5.36	37.5±5.05	36.1±3.41	0.623
MCV (fL)	83.0±9.00	84.2±7.89	82.3±7.80	0.791
Iron (µg/dL)	61.4±46.6	52.9±32.6	43.9±25.2	0.448
Transferrin (mg/dL)	287.0±48.8	290.4±52.6	313.9±63.9	0.407
TSI (%)	17.5±12.7	14.7±8.68	12,0±7.37	0.332
Ferritin (µg/L)	45.2±50.6^a	63.6±100.2^a	207.0±396.6^b	0.014
Tumor markers				
CEA (ng/mL)	6.65±11.2	7.47±10.5	14.0±13.2	0.380
CA 19.9 (IU/mL)	23.5±30.1^a	22.1±10.5^a	106.7±169.8^b	0.003

Data are expressed as mean ± standard deviation. Values with different superscript letters (**a**, **b**) were significantly different when the 3 groups were compared by one-way ANOVA followed by a Student–Newman-Keuls post hoc test. IL-6: Interleukin 6; CRP: C-reactive protein; N/L: neutrophil/lymphocyte ratio; MCV: mean corpuscular volume; TSI: transferrin saturation index; CEA: carcinoembryonic antigen; CA 19.9: carbohydrate antigen 19.9.

Supplementary Figure S1. Flow chart of the study protocol.

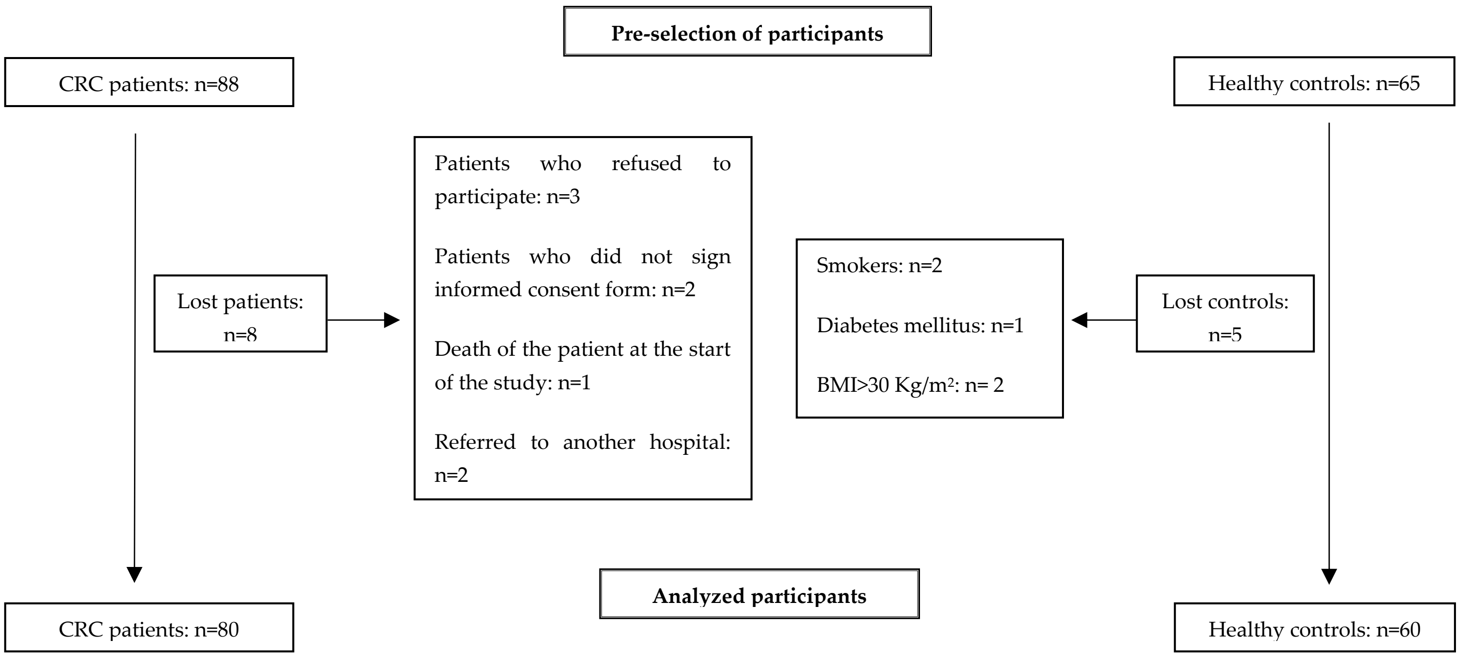

Supplement: Supplementary file 1 [file ijms-23-11664-s001.zip › ijms-1935541-supplementary.pdf]
